# Spintronic terahertz emission with manipulated polarization (STEMP)

**DOI:** 10.1007/s12200-022-00011-w

**Published:** 2022-04-21

**Authors:** Peiyan Li, Shaojie Liu, Xinhou Chen, Chunyan Geng, Xiaojun Wu

**Affiliations:** 1 School of Electronic and Information Engineering, Beihang University, Beijing, 100191 China; 2 School of Cyber Science and Technology, Beihang University, Beijing, 100191 China; 3 Wuhan National Laboratory for Optoelectronics, Huazhong University of Science and Technology, Wuhan, 430074 China

**Keywords:** Terahertz (THz) emission, Femtosecond laser, Spintronics, Polarization manipulation

## Abstract

Highly efficient generation and arbitrary manipulation of spin-polarized terahertz (THz) radiation will enable chiral lightwave driven quantum nonequilibrium state regulation, induce new electronic structures, consequently provide a powerful experimental tool for investigation of nonlinear THz optics and extreme THz science and applications. THz circular dichromic spectroscopy, ultrafast electron bunch manipulation, as well as THz imaging, sensing, and telecommunication, also need chiral THz waves. Here we review optical generation of circularly-polarized THz radiation but focus on recently emerged polarization tunable spintronic THz emission techniques, which possess many advantages of ultra-broadband, high efficiency, low cost, easy for integration and so on. We believe that chiral THz sources based on the combination of electron spin, ultrafast optical techniques and material structure engineering will accelerate the development of THz science and applications.

**Graphical Abstract:**

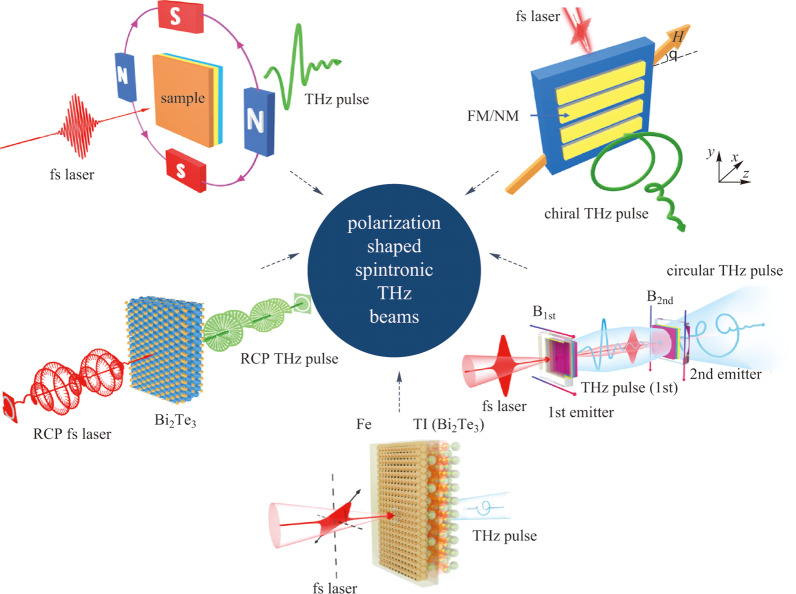

## Why chiral THz wave

**Fig. 1:**
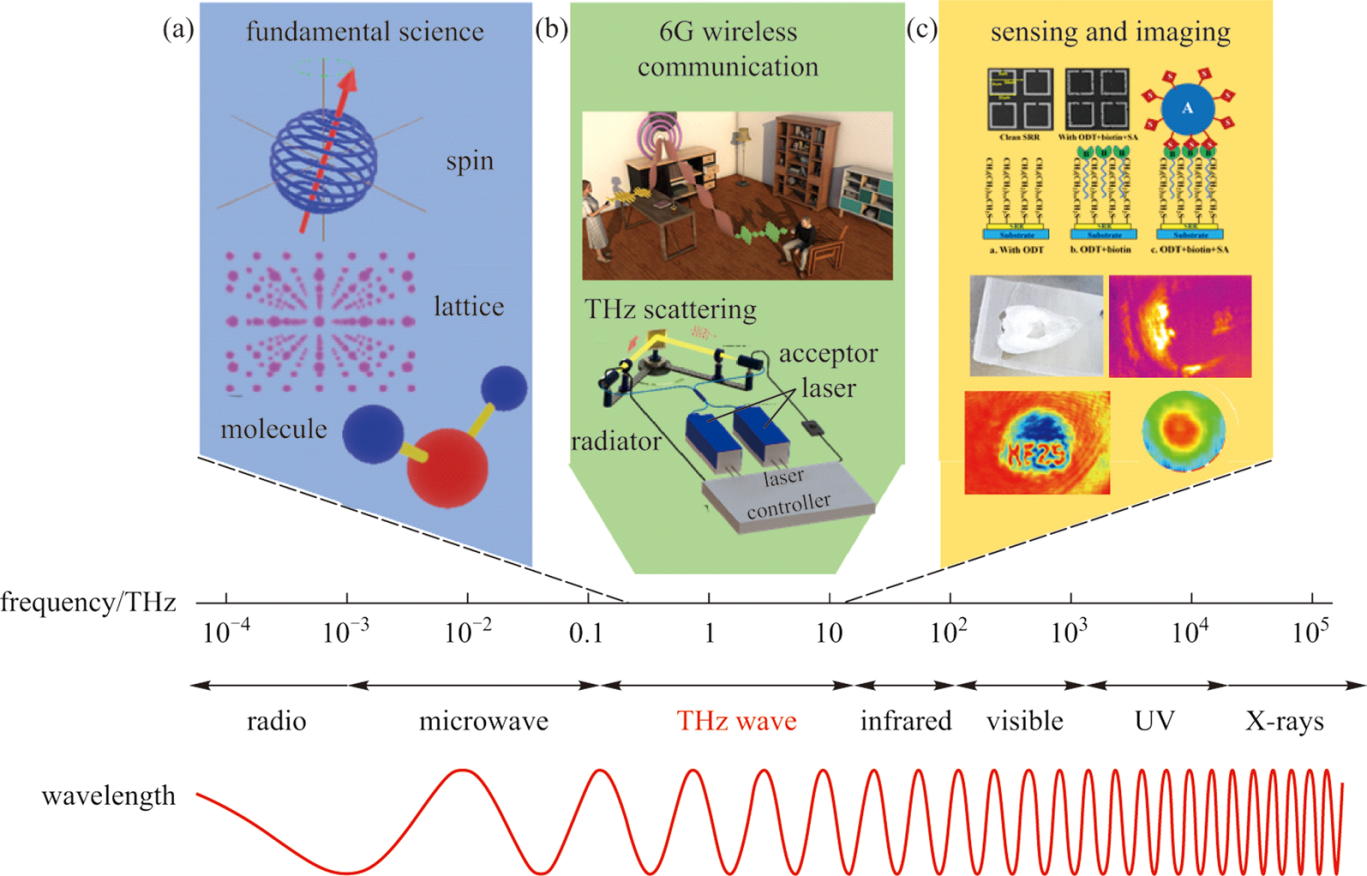
Location of THz frequency range and its promising disruptive applications. **a** Applications of THz spectroscopy in fundamental sciences. **b** THz band has been recognized as the carrier frequency for next-generation wireless communication. **c** THz sensing and imaging techniques enable practical applications in industry and biology. Adapted with permission from Refs. [30, 31]

The exact frequency range of terahertz (THz) electromagnetic wave, with its specific frequencies crossing between millimeter-wave and near-infrared, as illustrated in Fig. 1, is still controversial up to now. Some people think it is 0.1−10 THz, while others treat it as 0.3–30 THz. The electromagnetic wave in this special frequency band has many unique characteristics, having been predicted to possess many potential applications in fundamental sciences (Fig. 1a), wireless communication (Fig. 1b), sensing and imaging (Fig. 1c), and particle acceleration [1–31]. Hindering the reproduction of THz techniques lies in the shortage of highly efficient THz sources [2], sensitive THz detectors [3], and various functional devices [32–35], as well as cost-effective integrated THz systems [1, 5–7, 27, 29].

**Fig. 2:**
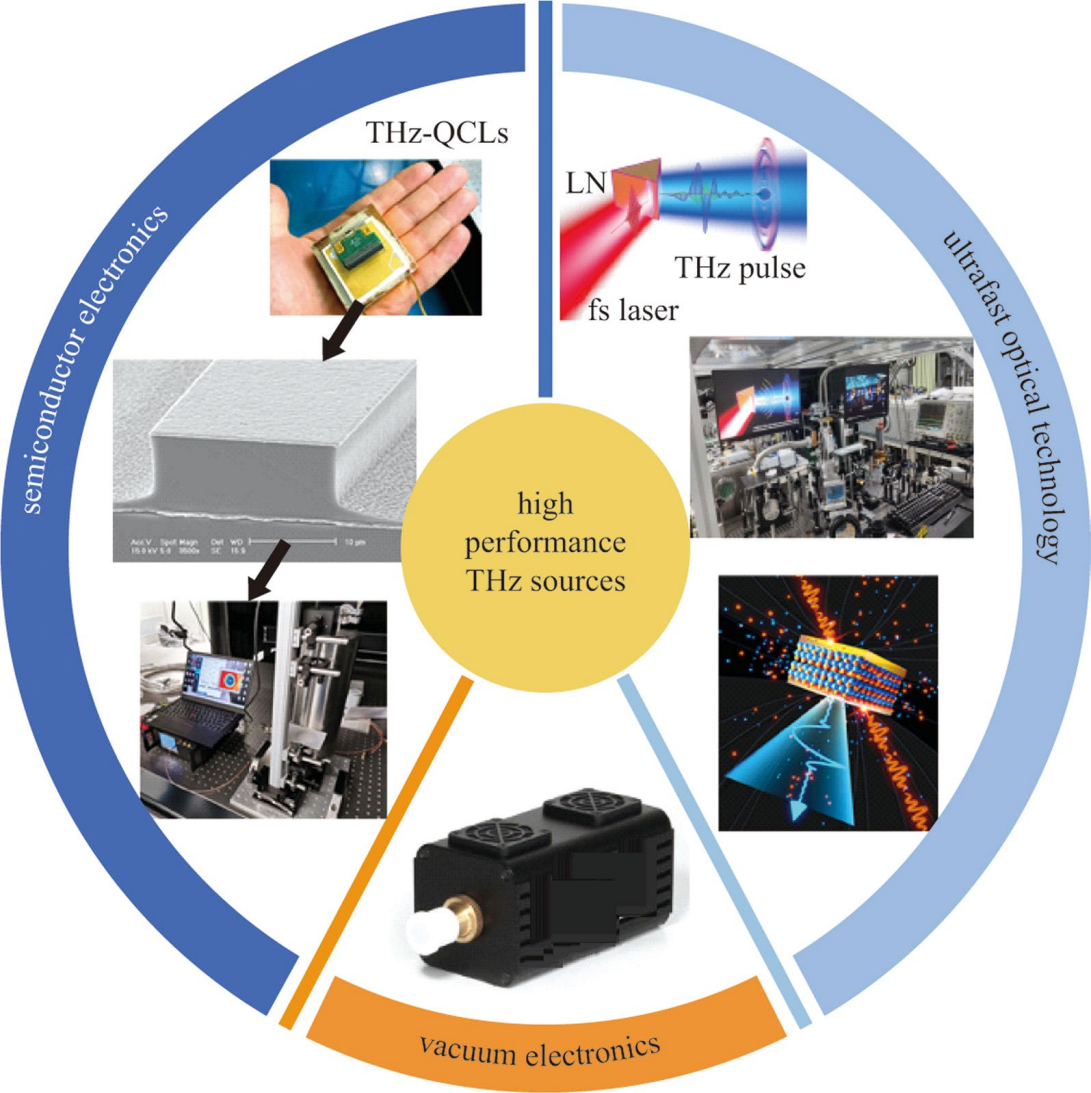
High-performance THz sources including commercialized vacuum electronic sources, semiconductor electronic quantum cascaded lasers and femtosecond laser-driven THz sources. Adapted with permission from Refs. [36, 55, 56]

Figure 2 summarizes various THz radiation sources including those preferred in laboratories and other commercialized THz emitters for practical applications. Along with rapid progress in ultrafast femtosecond laser technology, millijoule-level THz picosecond pulses not only enable the realization of table-top THz electron accelerators for compact X-ray sources from THz electron acceleration concept [11–21, 36], but also expect that THz biological effects will change from passive detection to active intervention [37–39], so as to realize treatment. Intense THz sources will also accelerate the frontiers of THz physics from linear to nonlinear regime for revealing more new phenomena and science yet been observed [40–49]. From the practical application facets [50–54], recent rapid progresses in semiconductor electronics enable chip-scale THz quantum cascaded lasers soon being possible to work at room temperature [55]. Meanwhile, vacuum electronic THz sources with compact and high average power output have been developed. Besides, employing the ultrafast cutting off behavior in electric field driven air nanoplasma formed inside nanogaps has been demonstrated to have the feasibility of delivering more than several hundred microwatts THz radiation with ultracompact and flexible advanced electronic characteristics [2]. These technological advances have laid the foundation for accelerating the application of THz in science and technology. However, compared with the pursuit of high efficiency, high power and high energy THz generation, how to realize controllable polarization states so as to manage both electric field amplitude as well as its temporal field vector has become an unavoidable challenge in THz optics.

Compared with the optical frequency band, the usage and management of THz polarization states are very backward. However, no less than the optical frequency band, polarization state tuned, especially circularly polarized THz electromagnetic wave is very useful in THz regime. For example, many quantum states of condensed matter and many biological macromolecules related to life have chiral characteristics [7, 17]. Circular polarization THz electromagnetic waves may also be required for THz wireless communication in the future. THz circular dichroism spectroscopy has been developed based on THz circular polarization obtained from chiral metasurfaces [7]. Non-relativistic manipulation of electron bunches has been realized by using strong-field THz circular polarization [8]. The spingalvanic effect was found by using circularly polarized THz waves in the 1990s [57]. These advances show that circularly polarized THz radiation is very important [58, 59], but at present, the progress is very slow.

**Fig. 3:**
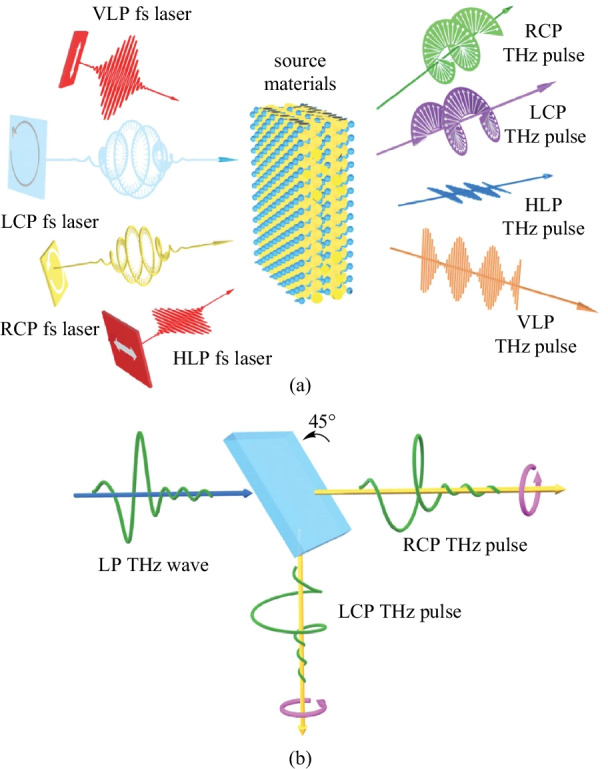
Twisting THz at the source vs conventional THz polarization manipulation. **a** Concept of integrating polarization manipulation at the THz sources. Adapted with permission from Ref. [66]. **b** Conventional separate scheme to realize THz polarization control. RCP: right-handed circular polarization; LCP: lefthanded circular polarization; HLP: horizontal linear polarization; VLP: vertical linear polarization; LP: linear polarization

Conventional methods of generating circularly polarized THz were learned from the optical frequency band. Therefore, the THz quarter-wave plate was invented by using the birefringence effect of quartz [60]. In recent years, with the emergence of metamaterials and metasurfaces, THz polarization transformation can also be realized by using periodic artificial structural materials [61–65]. However, these devices either have low conversion efficiency or narrow working bandwidth, which is not particularly convenient to use. If the function of THz polarization regulation can, as plotted in Fig. 3a, be integrated onto the source to realize twisting THz at the source, it would be expected to solve this problem in essence and in a more elegant way when comparing with those separate devices (see Fig. 3b).

**Fig. 4:**
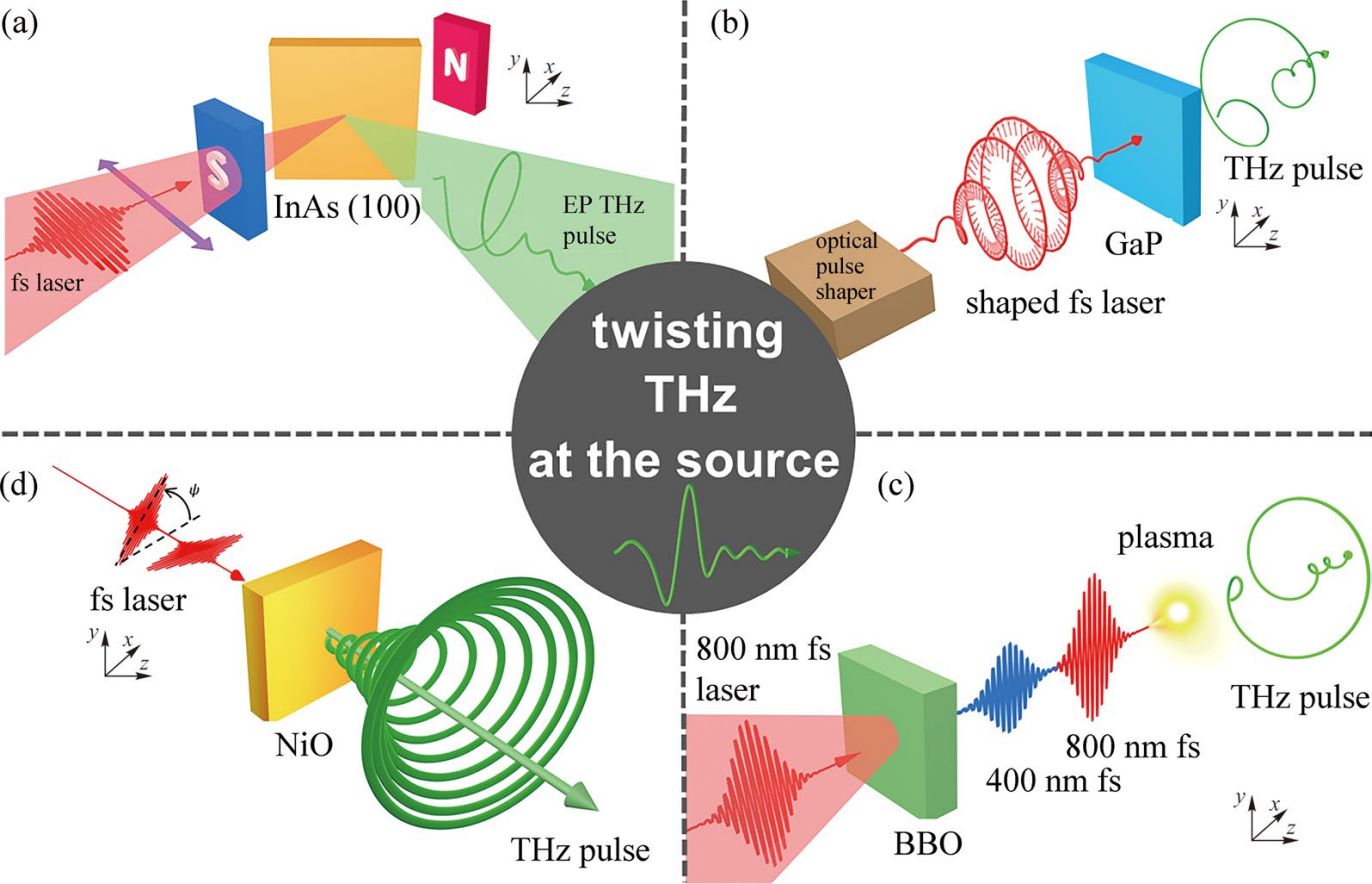
Examples of polarization tunable THz sources. **a** Strong magnetic field-controlled THz polarization in InAs emission scheme. Adapted with permission from Ref. [67]. **b** Optical pulse shaping can manipulate THz polarization from GaP crystals. Adapted with permission from Ref [72]. **c** Two-color pumped air plasma for arbitrary THz polarization generation. Adapted with permission from Refs [68, 69]. **d** Circularly polarized THz radiation from NiO crystals. Adapted with permission from Ref. [73]

In recent years, with the development of THz emission technology, there have been some cases of integrating the function of circular polarization generation and regulation at THz sources which have been concluded in Fig. 4. InAs THz sources, as shown in Fig. 4a, working in a reflection mode can realize elliptical THz emission and chiral regulation by adding a Tesla level strong magnetic field [67]. Later developed laser-driven two-color air plasma THz sources, as exhibited in Fig. 4c, can produce circularly polarized THz radiation by adding a helical electric field or magnetic field, and realize the arbitrary regulation of THz chirality via delicately tailoring the two fundamental and double frequency optical pumping pulses [68–71]. Using the combination of optical pulse shaper and THz generation mechanism of nonlinear crystals with special crystal structures (see Fig. 4b), the polarization characteristics of optical pumping light can be effectively transferred to the generated THz pulses, so as to realize the generation and regulation of circularly polarized THz radiation at the source [72]. More interesting, using the intrinsic magnon resonance at 1 THz frequency of antiferromagnetic NiO single crystal materials depicted in Fig. 4d, high-quality circularly polarized THz pulses have been demonstrated by controlling the time delay and linear polarization state of the two optical pumping pulses, and the THz chirality and ellipticity can be well manipulated [73]. These experimental results of generation and effective manipulation of circularly polarized THz radiation at the sources prove that the idea of realizing functionalized THz radiation at sources is feasible and very promising [25, 74]. Whether there are other THz light sources with better performance can also make good use of this idea, which is worthy of in-depth study, especially recently development spintronic THz emitters which were declared that it could not realize circularly polarized THz emission at its debut due to its working principle of inverse spin Hall effect (ISHE).

## Spintronic THz emission spectroscopy

**Fig. 5:**
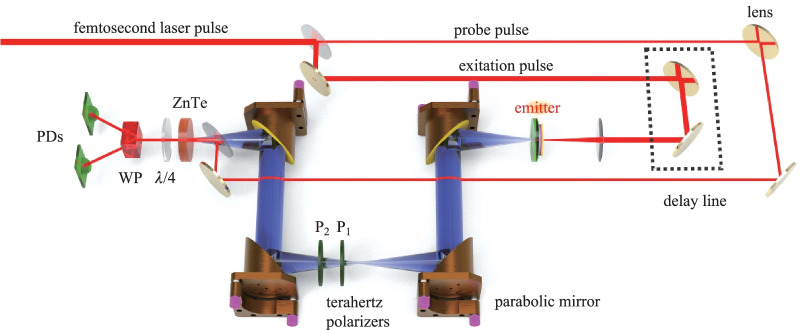
Schematic diagram of typical THz emission spectroscopy working in transmission measurement mode. Adapted with permission from Ref. [81]

Spintronic THz emission measurements can be directly implemented on the conventional THz time-domain spectrometer driven either by femtosecond laser oscillators or amplifiers [75–80]. Typical THz emission spectrometer working in transmission mode is schematically illustrated in Fig. 5. A femtosecond laser pulse is divided into two beams. One for excitation ferromagnetic heterostructures and generate THz radiation, which is collimated and then focused. For polarization characterization, two THz polarizers are inserted into the THz beam path. The first-polarizer is aligned according to the arrangement of the (110) ZnTe detection crystal to ensure THz signals are detectable, while the second one is automatically rotated with a  ± 45° to resolve the THz polarization state. The transmitted THz waves after the two polarizers are then further collimated and focused together with the probe pulse by a pellicle onto the ZnTe crystal. The THz electric field vector is recorded by the electro-optical sampling system which includes a quarter-wave plate, a Wollaston prism, and two photodiodes [81–84].

**Fig. 6:**
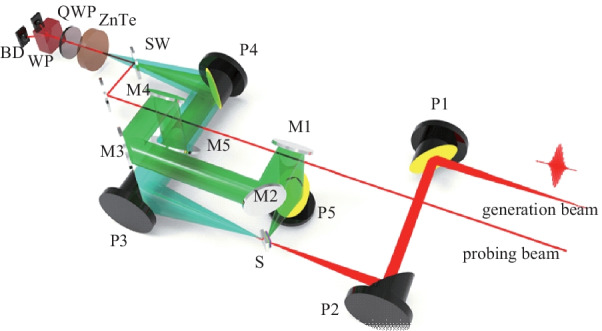
Incident angle resolved spintronic THz emission spectroscopy with both transmission and reflection working modes. Adapted with permission from Ref. [85]

Sometimes, reflection measurements from spintronic THz emitters are also required. As illustrated in Fig. 6, an incident angle resolved THz transmission and reflection spectrometer can be very useful [85]. This kind of THz emission spectrometer is more complicated than that shown in Fig. 5. When femtosecond laser pulses illuminate onto the ferromagnetic heterostructure, the radiation THz signal in the transmission direction is collimated and focused by P3 and P4, when the Al reflection mirror is removed from the THz path. The reflected THz signal is first collimated by P5, and then goes through M1–M5, and finally is focused by P4 onto the ZnTe detection crystal. The reflective THz emission spectroscopy is widely applicable for investigating THz emission physics from bulk quantum materials, which is difficult to be fabricated into nanofilms [83].

## How to use electron spin to generate THz radiation

**Fig. 7:**
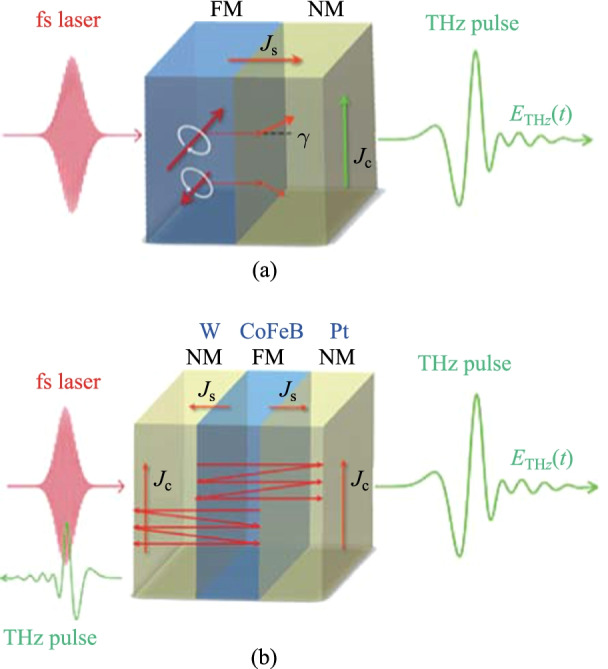
Schematic diagram of ferromagnetic metal/nonmagnetic metal (FM/NM) heterostructures for radiating THz pulses. **a** Fs laser pulses illuminate onto FMs and transfer the optical energy to electrons. Spin up and spin down electrons are excited above Fermi level and start moving. Due to spin-to-orbital coupling, longitudinal spin polarized currents will convert to plane charge current and radiate THz waves. **b** Enhanced THz radiation is observed when W layer is attached onto FM. Adapted with permission from Ref. [89]

In fact, spintronic THz emission originated from the curiosity of the original physics of ultrafast demagnetization. When the development of ultrafast demagnetization and ultrafast THz optics crossed, Beaureparie et al. obtained THz radiation from iron nanofilm in 2004 [86]. But the signal was very weak at that time. They interpreted this phenomenon as magnetic dipole radiation in the process of femtosecond demagnetization process. After 6 years, Battiato et al. proposed that the formation of longitudinal ultrafast spin current was the physical mechanism of ultrafast demagnetization [87]. Inspired by this idea, in 2013, Kampfrath and his coworkers covered a layer of conventional heavy metal Au or Ru onto the Fe ferromagnetic nanofilm, and realized the conversion of longitudinal spin current to transverse in-plane charge current through ISHE [88]. Figure 7a plots the schematic diagram of the ISHE principle. Through THz emission spectroscopy, they indirectly detected the spin dynamics on the femtosecond time scale. Through in-depth analysis of the experimental data, they found that this method can produce a very large current density between the ferromagnetic metal (FM) and the nonmagnetic metal (NM), which was expected to achieve a high-efficiency THz radiation source through the improvement of materials and structures.

In 2016, after comprehensive investigation of more than 70 FMs and NMs as well as their heterostructures, this group realized efficient and ultra-broadband spintronic THz emitters [89]. According to the formula of ISHE,

1 $$\overrightarrow{E} {{_{{{\text{THz}}}} }} \propto \overrightarrow {{J_{{\text{c}}} }} { = }\gamma \overrightarrow {{J_{{\text{s}}} }} \times \frac{{\overrightarrow {M} }}{{\ {\left| \overrightarrow{M} \right|} }},$$

where $\overrightarrow{E} {{_{{{\text{THz}}}} }}$ is the radiated THz electric field vector, $\overrightarrow {{J_{{\text{c}}} }}$ is the spin converted transverse charge current, $\gamma$ is the spin Hall angle for W and Pt, $\overrightarrow {{J_{{\text{s}}} }}$ is the light induced spin current, and $\overrightarrow {M}$ is the magnetization, in order to obtain highly efficient THz emission, ferromagnetic materials with high light absorption efficiency need to be selected [90–92], because it can ensure to provide enough high-density spin electrons. Heavy metals with large hall angles should be chosen because they can ensure higher efficiency of spin to charge conversion. Interestingly, in order not to waste the spin current generated from the back direction, as shown in Fig. 7b, the coherent superposition of THz radiation generated by the two interfaces can be realized by using two heavy metals with opposite spin Hall angles, which greatly improves the THz emission efficiency [89]. This report shows that under 10 fs ultrashort laser pumping, the three-layer W/CoFeB/Pt with a total thickness of 5.8 nm had the capability of producing THz radiation more than commercial nonlinear crystals and photoconductive antennas. The bandwidth of the spintronic emitter is only determined by the laser pulse width, so the spin emitter pumped by 10 fs can obtain an ultrabroad bandwidth of 1–30 THz.

**Fig. 8:**
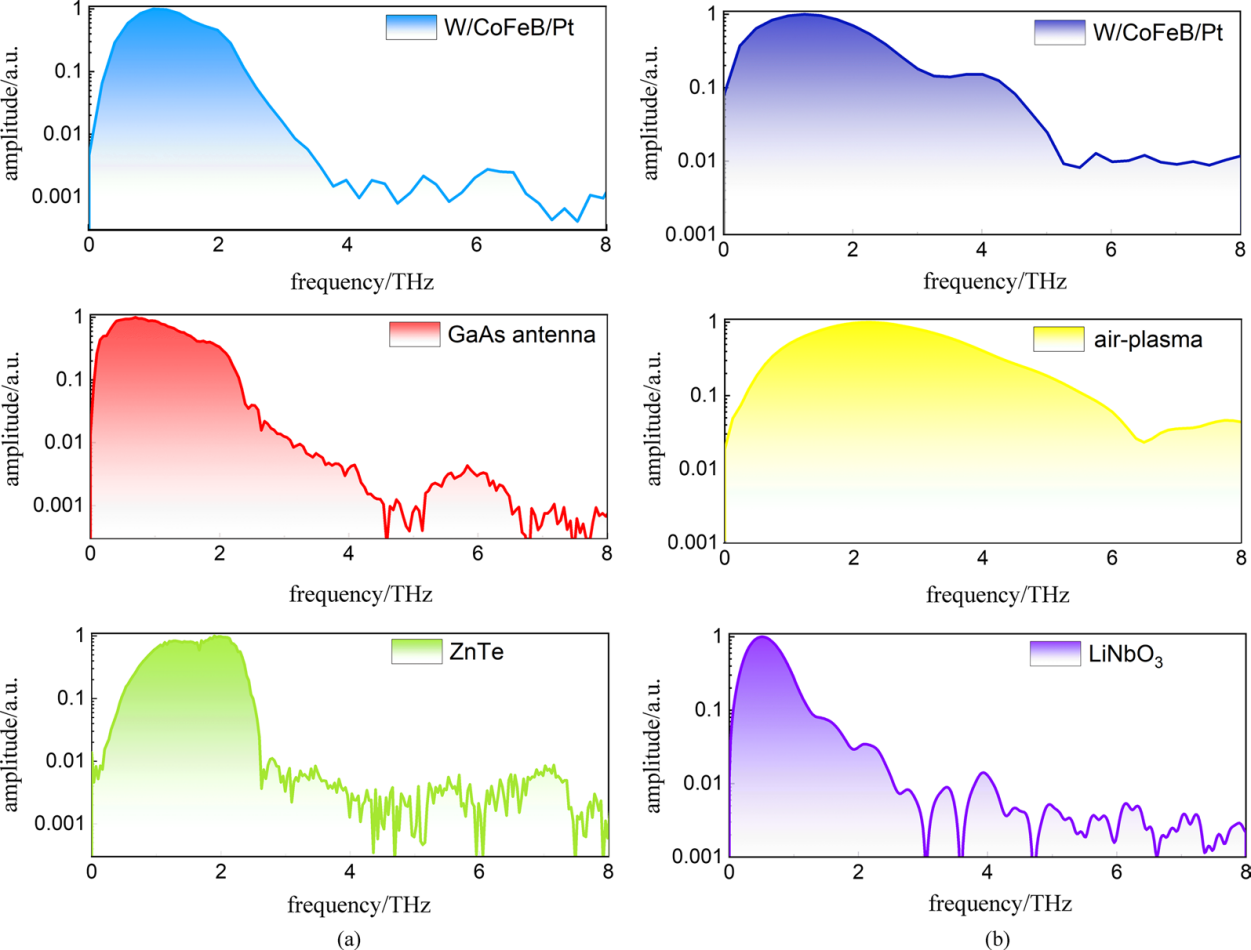
Radiated THz spectrum comparison from several emitters driven by **a** typical femtosecond laser oscillator, and **b** femtosecond laser amplifier

Figure 8a summarizes several THz radiation spectra from spintronic THz emitters with W(1.8 nm)/CoFeB(1.8 nm)/Pt(1.8 nm), commercial low-temperature grown GaAs antennas with a 10 μm gap, and a 1-mm thick ZnTe nonlinear crystal, all of which are generated by a femtosecond laser oscillator with 800 nm central wavelength, 100 fs pulse duration, and 80 MHz repetition rate. Under the same laser pumping, spintronic THz emitters have relatively broader spectrum than that from ZnTe and are equivalent to that from GaAs antenna with 10 μm gap.

Figure 8b plots the THz spectrum comparison among spintronic emitters of W(1.8 nm)/CoFeB(1.8 nm)/Pt(1.8 nm), air-plasma radiation, and lithium niobate crystals with tilted pulse front techniques. All these spectra are obtained based on femtosecond laser amplifiers. For the first two, we employ a Ti:sapphire femtosecond laser amplifier with a central wavelength of 800 nm, pulse duration of 35 fs, and repetition rate of 1 kHz, while the tilted pulse front technique is obtained on a 10-Hz, 30-fs, 800-nm amplifier. When comparing with air-plasma THz sources, the spintronic THz emitters have equivalent bandwidth ~ 6 THz, which is much broader than that from LiNbO_3_.

Besides, strong-field THz generation with 300 kV/cm field strength from 5.6 nm-thick W/CoFeB/Pt heterostructures was also demonstrated with 1–10 THz bandwidth under the excitation of a 5.5-mJ, 40-fs, 800-nm femtosecond laser amplifier system [93]. These pioneering works have opened the door to the research of ultrafast spin THz optoelectronics, and follow-up work emerges one after another.

**Fig. 9:**
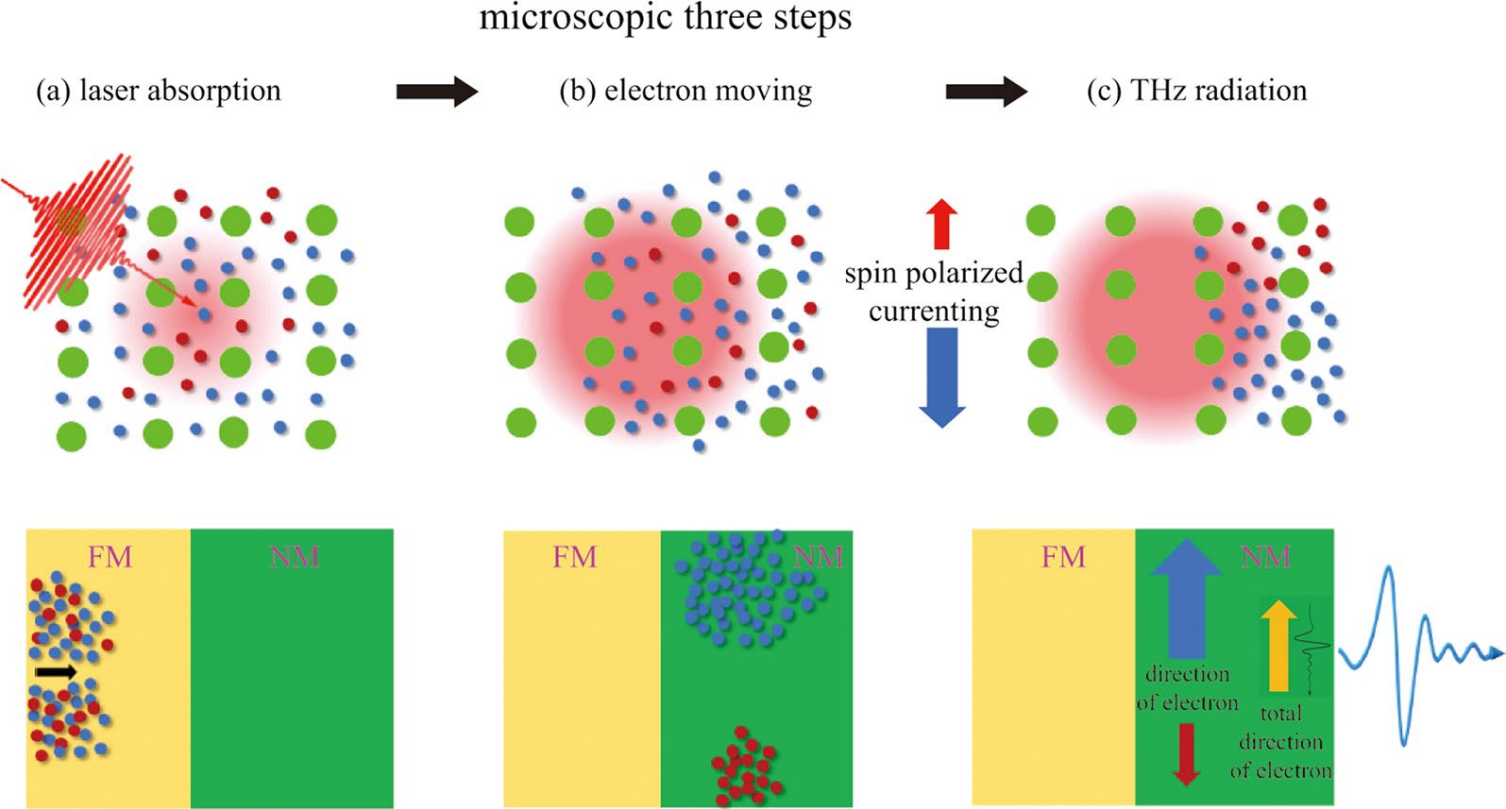
Microscopic three steps for spintronic THz emission: **a** laser absorption, and **b** electron moving, and **c** THz radiation

Understanding from a microscopic picture, the spintronic THz emission process can, as illustrated in Fig. 9, be divided into three steps: (1) laser absorption, (2) electron moving, and (3) THz radiation. In the process, there exists electron scattering induced magnetization quenching. The whole qualitative description of the microscopic three steps can be explained in the following. When ultrafast laser pulses with a time-varying profile in the femtosecond time scale illuminate onto a FM/NM heterostructure, the FM absorbs the light energy and the electron spins are excited below the Fermi level into above Fermi level. Due to the differences of the density, band velocity and mobility of the spin up and spin down electrons, a longitudinal spin polarized current is formed along the propagation direction of the pumping laser pulse. When this spin current flows into the heavy metal, it will be converted to net transverse charge current due to ISHE which originates from the spin–orbit coupling. Such current possesses a femtosecond varying property, and it radiates THz pulses according to the Maxwell equation [94–96].

## Helicity dependent THz spin currents

For spintronic THz emission, according to ISHE, the generated THz polarity should be always perpendicular to the external applied magnetic field direction. What is more interesting is that the THz radiation properties manifest insensitive to the pumping laser polarization states. Therefore, it is difficult to control the emitted THz waves as well as the ultrafast spin current. In ultrafast spintronics, it is significant in highly efficient injection of spin electrons, ultrasensitive detection and effective manipulation of ultrafast spin currents. In the spintronic THz emission process, ultrafast injection is realized via femtosecond laser pulses, while the ultrasensitive coherent detection of THz spin currents via indirect THz emission spectroscopy. The left challenge is how to realize effective control of the femtosecond spin current.

**Fig. 10:**
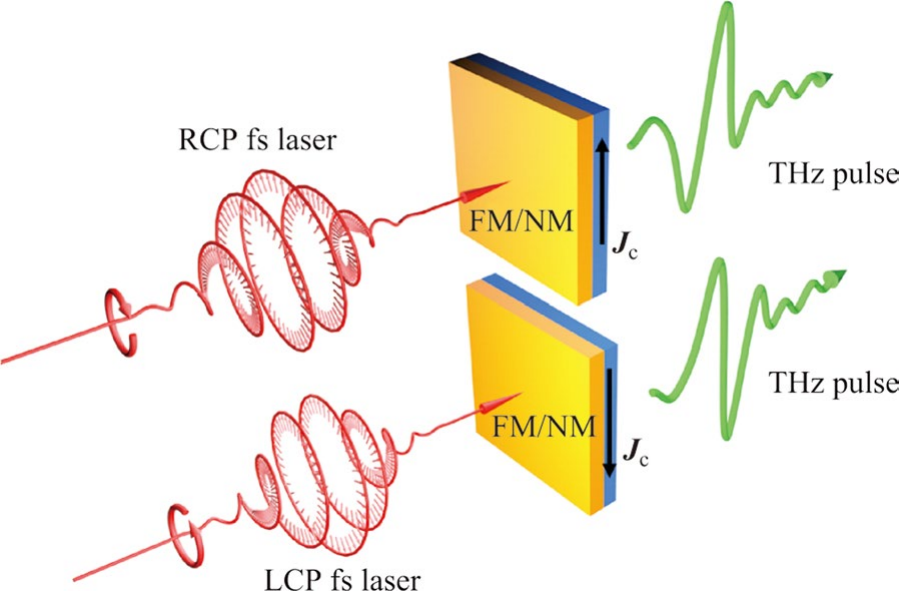
Pumping laser helicity dependent spintronic THz radiation. The radiated THz polarity can be controlled by the incident fs laser helicity. Adapted with permission from Ref. [90]

In 2016, Huisman et al. demonstrated that radiated THz polarity can be reversed by switching the pumping laser helicity via spin-orbital torque effect [90], as illustrated in Fig. 10. A similar phenomenon was also observed in the Rashba interface and the THz radiation symmetry can be broken by varying the pumping laser polarization states [77]. Both works are based on the spin–orbit interaction, under which circumstances, circularly polarized light can be used to induce helicity-dependent photocurrents due to the Rashba effect. This phenomenon indicates that the electron momentum and spin can be effectively coupled. The lack of space inversion symmetry in a conduction electron system, the spin–orbit interaction can result in the degeneracy breaking for spin-up and spin-down electron sub-bands, which can lead to the formation of spin–orbit torque. A tilting magnetization can be induced and forms an electric current as an inverse effect, named inverse spin–orbit torque tilting. Therefore, circularly polarized light can be employed to tilt the magnetization. These phenomena provide an effective way to manipulate the THz spin current direction inside heterostructures, but the realization of arbitrarily controlling the spin current vector in a more complex way has not yet been obtained.

## Polarization management in spintronic THz emitters

**Fig. 11:**
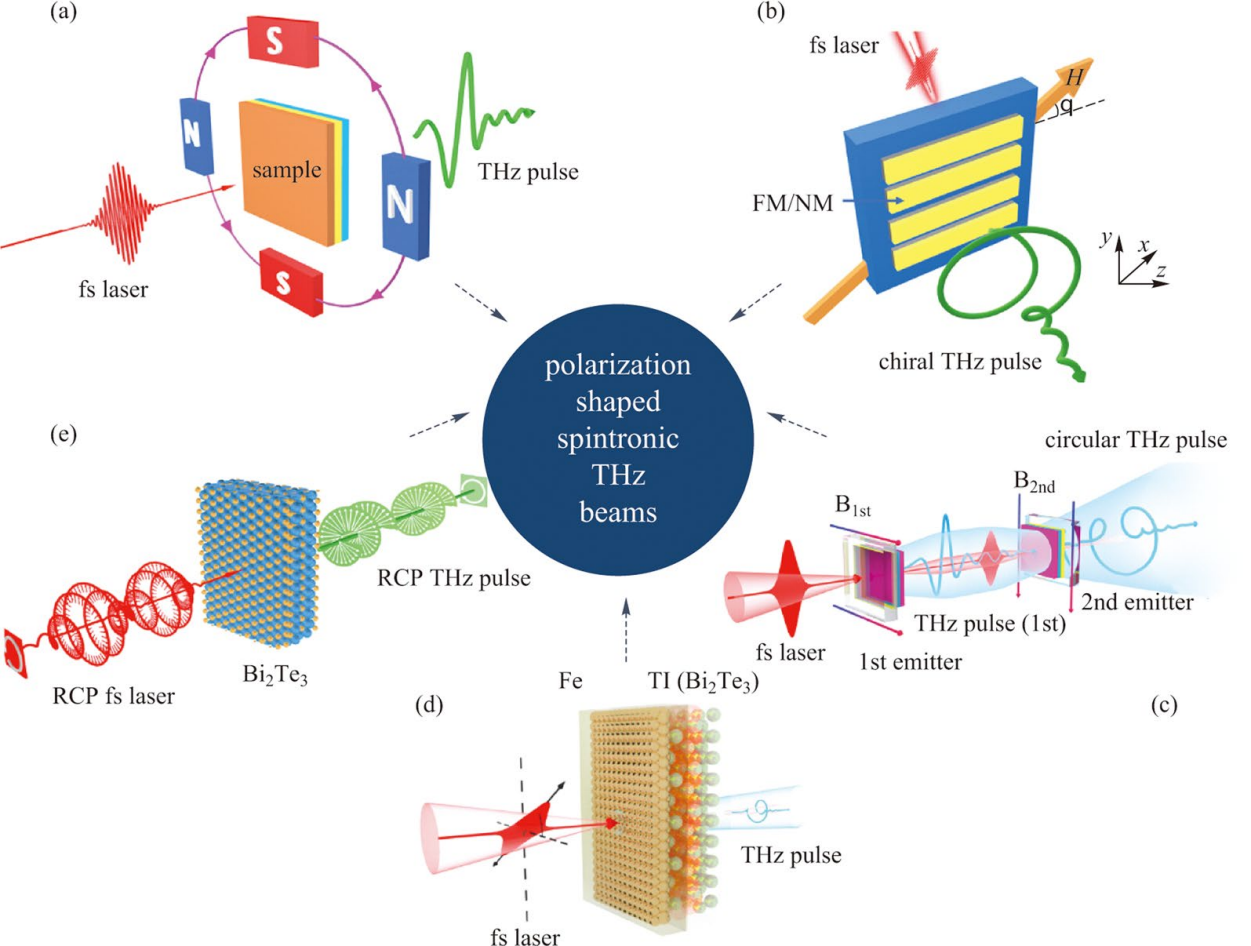
Polarization tunable spintronic THz emitters. **a** Arbitrary manipulation of THz polarization by controlling the external applied magnetic field distribution. Adapted with permission from Refs. [81, 98, 99]. **b** Integrating metastructures with FM/NM heterostructures for circularly polarized THz. Adapted with permission from Ref. [97]. **c** Cascaded THz radiation scheme for circularly polarized THz generation. Adapted with permission from Ref. [82]. **d** Elliptically polarized THz from the topological insulator (TI)|iron (Fe) heterostructure. Adapted with permission from Ref. [56]. **e** Polarization tunable THz from the topological insulator Bi_2_Te_3_ controlled by fs laser polarization and sample azimuthal angles. Adapted with permission from Ref. [66]

Polarization shaping with prescribed manipulation of the temporal evolution of the amplitude and direction of electric field vectors for spintronic THz pulses is very challenging. Up to now, as summarized in Fig. 11, people have reported several effective ways to manipulate THz polarization states including artificial structure integrated methods [97], external magnetic field distribution tailoring [81, 98, 99], cascaded radiation scheme [82], novel heterostructures and optical methods [56, 66]. The following parts will introduce these approaches one by one.

In 2016, Prof. Qi’s group proposed a stripe structure to engineer the radiated THz pulses and THz amplitudes can be modulated as exhibited in Fig. 11a [100]. Recently, Prof. Tao’s group collaborate with Prof. Zhou’s group and proposed a hybrid spintronic THz emitter integrated with metasurfaces (see Fig. 11b) [97]. This is a very effective way to combine both advantages of spintronic THz emitters and THz metasurfaces. The former has the merits of ultra-broadband, low cost, high efficiency and easy to integrate, while the latter has been proved to hold the capability of producing high-quality spin-polarized THz beams. Taking the active spintronic-metasurface THz emitters for tunable THz chirality as an example, the effective interaction between light and matter has played an irreplaceable role in this process. The coupling between the femtosecond laser-induced spintronic currents and the metasurface-induced ultrafast currents is employed to tune the THz chirality.

If we go back and carefully analyze the ISHE formula, it is not difficult to find that the polarization characteristics of THz radiation primarily depend on the properties of the applied magnetic field. To obtain high-quality circularly polarized THz radiation, we need to meet three conditions: (1) generating two THz electric field components with their polarization perpendicular to each other; (2) their amplitudes should be equal or close; (3) their phase difference needs to be 90 degrees. In our group, we delicately tailored the applied magnetic field distribution, and easily produced elliptically polarized THz pulse with its arbitrarily tuned chirality, ellipticity and principle axis [81]. With similar methods, a quadrupole-like THz polarization profile was also demonstrated in spintronic THz emitters via applying a specific magnetic field pattern to the source [99]. By coupling a triangular silicon prism, azimuthally- and radially-polarized THz cylindrical vector beams have been successfully demonstrated [98].

Interestingly, when femtosecond laser pulses interact with FM/NM heterostructures, although a very huge current density can be formed at the interface, there is still at least half of the pumping light energy left. If we could recycle these energies to further drive another sample, it would enable higher energy conversion efficiency as well as the feasibility of producing circularly polarized THz waves. Such an idea was realized in a cascaded geometry as shown in Fig. 11c [82]. In conventional spintronic THz emitters, the radiated THz waves are always linearly polarized and can be tuned by rotating the applied magnetic field directions.

**Fig. 12:**
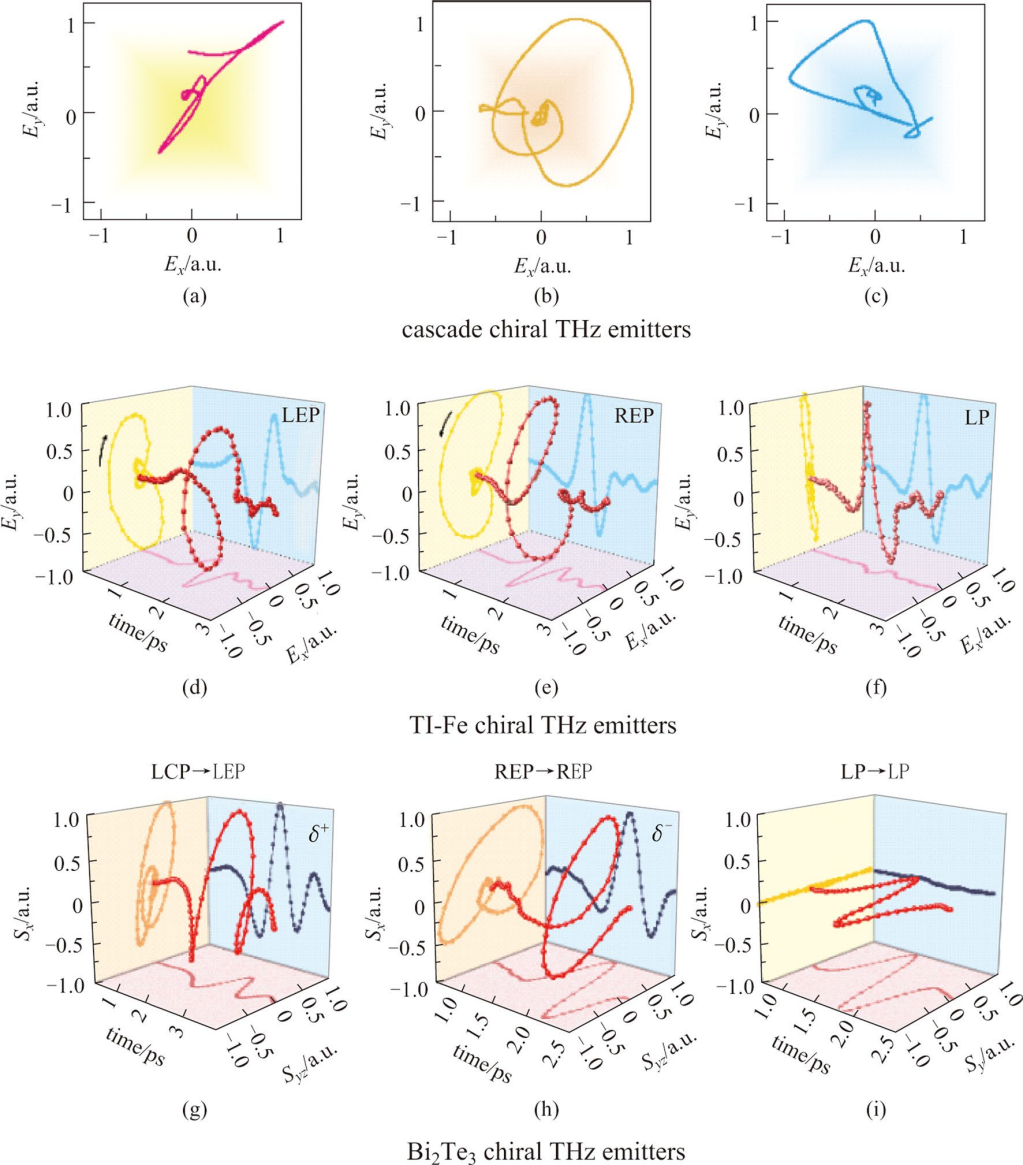
Experimentally produced chiral THz wave manipulation results from cascade method, TI-Fe chiral THz emitters and Bi_2_Te_3_ sources. **a** For cascade chiral THz emitters, when two parallel magnetic fields are arranged on two stages, it will lead to the generation of linearly polarized (LP) THz waves. **b** Circular polarization is obtained when applying vertical magnetic fields. **c** Adjusting the intersection angle can produce an elliptically polarized THz beam. For TI-Fe heterostructures, **d** left-handed, **e** right-handed elliptical polarizations (LEP and REP), and **f** linear polarization are manipulated by combining the spin-to-charge conversion (SCC) effect and linear photogalvanic effect (LPGE). **g**–**i** For Bi_2_Te_3_, the conversion between different chirality and various polarization state can be obtained by controlling the pumping laser polarization and sample azimuthal angles. Adapted with permission from Refs. [56, 66, 82]

According to Eq. (1), it can be inferred that the intersection angle between the two THz electric fields can be exactly controlled by that between the two applied magnetic field directions. Therefore, perpendicular electric field components were prepared by arranging the first and second magnetic field direction to be horizontal and vertical respectively, while the equal amplitudes for both fields were naturally satisfied for the second emitter also had an almost half percent absorption for the first THz pulse. The most intriguing part was the origin of the 90° phase difference between the two THz electric fields. It was also naturally obtained based on the tiny refractive index of the air inside the vacuum chamber between the THz wave and the residual optical pumping light when both of them propagated from the first emitter to the second one. Figure 12a–c gives the polarization manipulation results. As long as the magnetic field direction is accurately designed, the radiation THz polarization can be transformed from linear to circular and elliptical. When the direction of the magnetic field applied to the two emitters is parallel, a linearly polarized THz radiation can be obtained, as shown in Fig. 12a. When the direction is vertical, it becomes circular (Fig. 12b). When the two magnetic fields are not perpendicular or parallel, the THz wave is elliptical polarization. When the intersection angle of two magnetic field directions is 45°, the experimental results are shown in Fig. 12c. With this method, a high-quality circularly polarized THz beam was produced and its chirality can be easily manipulated by flexibly modulating the magnetic field distribution.

However, when we grew a Fe onto TI but illuminated this heterostructure by linearly polarized femtosecond laser pulses, we can also obtain high-quality THz waves with even much higher radiation efficiency [56]. As shown in Fig. 11d, we abandoned circular photogalvanic effect induced THz electric field component, and only employed linear photogalvanic effect induced shift current and spin-to-charge conversion effect induced charge current to combine chiral THz waves. The three requirements for generating chiral THz waves are: manipulating the magnetization azimuth or pumping laser polarization angle make the electric field of the two effects induced polarization state orthogonal. With the intrinsic 85.5° phase difference of the two effects, TI|Fe heterostructures can produce chiral THz radiation when excited by linearly polarized pumping laser pulses. The manipulation of ellipticity can be successfully initiated by varying the spatial polarization intersection angle between the electric field generated by linear photogalvanic effect and the electric field converted by spin-to-charge conversion effect. As has already been mentioned before, the two electric field components can be, respectively, modulated by the pumping light polarization angle and the magnetization azimuthal angle. As a consequence, there exist two kinds of specific ellipticity modulation methods. (1) Appling a fixed horizontal direction magnetic field which can make the magnetization azimuthal angle of spin-to-charge conversion effect completely orientate vertical direction, and further controlling the incident light polarization angle. (2) Fixing the incident polarization angle to orientate the component corresponding to linear photogalvanic effect along with the vertical direction, and rotating the applied magnetic field direction, indicating the ellipticity can be adjusted accordingly. The results of the produced THz waves of left-handed elliptic polarization, right-handed elliptic polarization and linear polarization are shown in Fig. 12d and e. For these experiments, samples were fabricated as ultrathin nanofilms. For some novel quantum materials with bulk state, reflective THz emission spectroscopy have been employed to study the ultrafast quantum phenomena and physics [28, 80]. Furthermore, THz emission spectroscopy has also been widely used to investigate light-matter interaction in two-dimensional materials and their heterostructures from macro- to micro-scales and then extending to nanoscale [6, 101].

The aforementioned three methods are relatively straightforward for manipulating the THz polarizations. Actually, we can also realize THz polarization shape engineering through the phase difference due to the time difference between different THz emission mechanisms inside topological materials and their heterostructures. Taking topological insulator Bi_2_Te_3_ as an example, when linearly polarized THz emission was obtained when the incident femtosecond laser was also linearly polarized [66]. However, radiated THz pulses became circularly polarized if we delicately control the sample azimuthal angle and use circularly polarized femtosecond laser pulses for pumping. The produced THz waves can be arbitrarily controlled by adjusting the chirality and ellipticity of the incident circularly polarized light. Helicity dependent current is the critical reason why we can obtain spin-polarized THz pulses because we can continuously tune the magnitude and its polarity by changing the helicity. Figure 11e illustrates that the generation and manipulation of chiral THz waves have been successfully realized in this TI through finely managing the time difference between linear and circular photogalvanic effects. The results of polarization manipulation are shown in Fig. 12g–i. The THz wave radiated from the Bi_2_Te_3_ chiral THz emitters is consistent with the chirality of the pumping laser.

## Conclusion and outlook

Spintronic THz emitters have been predicted to be one of the best candidates for the next-generation THz sources since they possess many distinct merits such as high efficiency, ultra-broadband, low cost, ultrathin, and easy for integration. However, there are still many challenges need to be overcome when pushing these materials to be used for the generation of high-field THz with convenient polarization manipulation. Furthermore, integration with fiber laser-based THz pulses or continuous wave systems will also accelerate the spintronic THz emitters to be easily adapted to industrial applications. Therefore, exploring new materials, fabricating new structures, further improving optical-to-THz energy conversion efficiency and further realizing functionalization at the radiation sources are expected to be applied, which is the direction of spintronic THz emission that needs further research.

## References

[1] Li Q, Stoica VA, Paściak M, Zhu Y, Yuan Y, Yang T, McCarter MR, Das S, Yadav AK, Park S, Dai C, Lee HJ, Ahn Y, Marks SD, Yu S, Kadlec C, Sato T, Hoffmann MC, Chollet M, Kozina ME, Nelson S, Zhu D, Walko DA, Lindenberg AM, Evans PG, Chen LQ, Ramesh R, Martin LW, Gopalan V, Freeland JW, Hlinka J, Wen H (2021). Subterahertz collective dynamics of polar vortices. Nature.

[2] Samizadeh Nikoo M, Jafari A, Perera N, Zhu M, Santoruvo G, Matioli E (2020). Nanoplasma-enabled picosecond switches for ultrafast electronics. Nature.

[3] Peng K, Jevtics D, Zhang F, Sterzl S, Damry DA, Rothmann MU, Guilhabert B, Strain MJ, Tan HH, Herz LM, Fu L, Dawson MD, Hurtado A, Jagadish C, Johnston MB (2020). Three-dimensional cross-nanowire networks recover full terahertz state. Science.

[4] Hafez HA, Kovalev S, Deinert JC, Mics Z, Green B, Awari N, Chen M, Germanskiy S, Lehnert U, Teichert J, Wang Z, Tielrooij KJ, Liu Z, Chen Z, Narita A, Müllen K, Bonn M, Gensch M, Turchinovich D (2018). Extremely efficient terahertz high-harmonic generation in graphene by hot Dirac fermions. Nature.

[5] Cocker TL, Jelic V, Hillenbrand R, Hegmann FA (2021). Nanoscale terahertz scanning probe microscopy. Nat. Photonics.

[6] Plankl M, Faria Junior PE, Mooshammer F, Siday T, Zizlsperger M, Sandner F, Schiegl F, Maier S, Huber MA, Gmitra M, Fabian J, Boland JL, Cocker TL, Huber R (2021). Subcycle contact-free nanoscopy of ultrafast interlayer transport in atomically thin heterostructures. Nat. Photonics.

[7] Choi WJ, Cheng G, Huang Z, Zhang S, Norris TB, Kotov NA (2019). Terahertz circular dichroism spectroscopy of biomaterials enabled by kirigami polarization modulators. Nat. Mater..

[8] Kovalev S, Hafez HA, Tielrooij KJ, Deinert JC, Ilyakov I, Awari N, Alcaraz D, Soundarapandian K, Saleta D, Germanskiy S, Chen M, Bawatna M, Green B, Koppens FHL, Mittendorff M, Bonn M, Gensch M, Turchinovich D (2021). Electrical tunability of terahertz nonlinearity in graphene. Sci. Adv..

[9] Jelic V, Iwaszczuk K, Nguyen PH, Rathje C, Hornig GJ, Sharum HM, Hoffman JR, Freeman MR, Hegmann FA (2017). Ultrafast terahertz control of extreme tunnel currents through single atoms on a silicon surface. Nat. Phys..

[10] Pistore V, Nong H, Vigneron PB, Garrasi K, Houver S, Li L, Giles Davies A, Linfield EH, Tignon J, Mangeney J, Colombelli R, Vitiello MS, Dhillon SS (2021). Millimeter wave photonics with terahertz semiconductor lasers. Nat. Commun..

[11] Xu H, Yan L, Du Y, Huang W, Tian Q, Li R, Liang Y, Gu S, Shi J, Tang C (2021). Cascaded high-gradient terahertz-driven acceleration of relativistic electron beams. Nat. Photonics.

[12] Kealhofer C, Schneider W, Ehberger D, Ryabov A, Krausz F, Baum P (2016). All-optical control and metrology of electron pulses. Science.

[13] Hibberd MT, Healy AL, Lake DS, Georgiadis V, Smith EJH, Finlay OJ, Pacey TH, Jones JK, Saveliev Y, Walsh DA, Snedden EW, Appleby RB, Burt G, Graham DM, Jamison SP (2020). Acceleration of relativistic beams using laser-generated terahertz pulses. Nat. Photonics.

[14] Zhang D, Fallahi A, Hemmer M, Wu X, Fakhari M, Hua Y, Cankaya H, Calendron AL, Zapata LE, Matlis NH, Kärtner FX (2018). Segmented terahertz electron accelerator and manipulator (STEAM). Nat. Photonics.

[15] Tang, H., Zhao, L., Zhu, P., Zou, X., Qi, J., Cheng, Y., Qiu, J., Hu, X., Song, W., Xiang, D., Zhang, J.: Stable and scalable multistage terahertz-driven particle accelerator. Phys. Rev. Lett. **127**(7), 074801–074807 (2021) 10.1103/PhysRevLett.127.074801 34459641

[16] Zhang, D., Fakhari, M., Cankaya, H., Calendron, A.L., Matlis, N.H., Kärtner, F.X.: Cascaded multicycle terahertz-driven ultrafast electron acceleration and manipulation. Phys. Rev. X **10**(1), 011067–011076 (2020)

[17] Zhao L, Wang Z, Tang H, Wang R, Cheng Y, Lu C, Jiang T, Zhu P, Hu L, Song W, Wang H, Qiu J, Kostin R, Jing C, Antipov S, Wang P, Qi J, Cheng Y, Xiang D, Zhang J (2019). Terahertz oscilloscope for recording time information of ultrashort electron beams. Phys. Rev. Lett..

[18] Zhang D, Fallahi A, Hemmer M, Ye H, Fakhari M, Hua Y, Cankaya H, Calendron AL, Zapata LE, Matlis NH, Kärtner FX (2019). Femtosecond phase control in high-field terahertz-driven ultrafast electron sources. Optica.

[19] Curry, E., Fabbri, S., Maxson, J., Musumeci, P., Gover, A.: Meter-scale terahertz-driven acceleration of a relativistic beam. Phys. Rev. Lett. **120**(9), 094801-094807 (2018) 10.1103/PhysRevLett.120.094801 29547316

[20] Ronny Huang W, Fallahi A, Wu X, Cankaya H, Calendron AL, Ravi K, Zhang D, Nanni EA, Hong KH, Kärtner FX (2016). Terahertz-driven all-optical electron gun. Optica.

[21] Nanni EA, Huang WR, Hong KH, Ravi K, Fallahi A, Moriena G, Miller RJ, Kärtner FX (2015). Terahertz-driven linear electron acceleration. Nat. Commun..

[22] Nova TF, Disa AS, Fechner M, Cavalleri A (2019). Metastable ferroelectricity in optically strained SrTiO_3_. Science.

[23] Sekiguchi, F., Hirori, H., Yumoto, G., Shimazaki, A., Nakamura, T., Wakamiya, A., Kanemitsu, Y.: Enhancing the hot-phonon bottleneck effect in a metal halide perovskite by terahertz phonon excitation. Phys. Rev. Lett. **126**(7), 077401–077407 (2021) 10.1103/PhysRevLett.126.077401 33666485

[24] Schmid CP, Weigl L, Grössing P, Junk V, Gorini C, Schlauderer S, Ito S, Meierhofer M, Hofmann N, Afanasiev D, Crewse J, Kokh KA, Tereshchenko OE, Güdde J, Evers F, Wilhelm J, Richter K, Höfer U, Huber R (2021). Tunable non-integer high-harmonic generation in a topological insulator. Nature.

[25] Keren-Zur S, Tal M, Fleischer S, Mittleman DM, Ellenbogen T (2019). Generation of spatiotemporally tailored terahertz wavepackets by nonlinear metasurfaces. Nat. Commun..

[26] Peller D, Kastner LZ, Buchner T, Roelcke C, Albrecht F, Moll N, Huber R, Repp J (2020). Sub-cycle atomic-scale forces coherently control a single-molecule switch. Nature.

[27] Li X, Qiu T, Zhang J, Baldini E, Lu J, Rappe AM, Nelson KA (2019). Terahertz field-induced ferroelectricity in quantum paraelectric SrTiO_3_. Science.

[28] Luo L, Cheng D, Song B, Wang LL, Vaswani C, Lozano PM, Gu G, Huang C, Kim RHJ, Liu Z, Park JM, Yao Y, Ho K, Perakis IE, Li Q, Wang J (2021). A light-induced phononic symmetry switch and giant dissipationless topological photocurrent in ZrTe_5_. Nat. Mater..

[29] Reimann J, Schlauderer S, Schmid CP, Langer F, Baierl S, Kokh KA, Tereshchenko OE, Kimura A, Lange C, Güdde J, Höfer U, Huber R (2018). Subcycle observation of lightwave-driven Dirac currents in a topological surface band. Nature.

[30] Wu, X., Quan, B., Pan, X., Xu, X., Lu, X., Gu, C., Wang, L.: Alkanethiol-functionalized terahertz metamaterial as label-free, highly-sensitive and specificbiosensor. Biosens. Bioelectron. **42**, 626–631 (2013) 10.1016/j.bios.2012.10.095 23261700

[31] Xiong, H., Cai, J., Zhang, W., Hu, J., Deng, Y., Miao, J., Tan, Z., Li, H., Cao, J., Wu, X.: Deep learning enhanced terahertz imaging of silkworm eggs development. iScience **24**(11), 103316–103329 (2021) 10.1016/j.isci.2021.103316 PMC8577140 34778731

[32] Pitchappa P, Kumar A, Prakash S, Jani H, Venkatesan T, Singh R (2019). Chalcogenide phase change material for active terahertz photonics. Adv. Mater..

[33] Vaswani, C., Mootz, M., Sundahl, C., Mudiyanselage, D.H., Kang, J.H., Yang, X., Cheng, D., Huang, C., Kim, R.H.J., Liu, Z., Luo, L., Perakis, I.E., Eom, C.B., Wang, J.: Terahertz second-harmonic generation from lightwave acceleration of symmetry-breaking nonlinear supercurrents. Phys. Rev. Lett. **124**(20), 207003–207009 (2020) 10.1103/PhysRevLett.124.207003 32501057

[34] Kang T, Kim RHJ, Choi G, Lee J, Park H, Jeon H, Park CH, Kim DS (2018). Terahertz rectification in ring-shaped quantum barriers. Nat. Commun..

[35] Dong T, Li S, Manjappa M, Yang P, Zhou J, Kong D, Quan B, Chen X, Ouyang C, Dai F, Han J, Ouyang C, Zhang X, Li J, Li Y, Miao J, Li Y, Wang L, Singh R, Zhang W, Wu X (2021). Nonlinear THz-nano metasurfaces. Adv. Funct. Mater..

[36] Zhang B, Ma Z, Ma J, Wu X, Ouyang C, Kong D, Hong T, Wang X, Yang P, Chen L, Li Y, Zhang J (2021). 1.4-mJ high energy terahertz radiation from lithium niobates. Laser Photonics Rev.

[37] Li Y, Chang C, Zhu Z, Sun L, Fan C (2021). Terahertz wave enhances permeability of the voltage-gated calcium channel. J. Am. Chem. Soc..

[38] Tachizaki T, Sakaguchi R, Terada S, Kamei KI, Hirori H (2020). Terahertz pulse-altered gene networks in human induced pluripotent stem cells. Opt. Lett..

[39] Liu X, Qiao Z, Chai Y, Zhu Z, Wu K, Ji W, Li D, Xiao Y, Mao L, Chang C, Wen Q, Song B, Shu Y (2021). Nonthermal and reversible control of neuronal signaling and behavior by midinfrared stimulation. Proc. Natl. Acad. Sci. U.S.A..

[40] Pein BC, Chang W, Hwang HY, Scherer J, Coropceanu I, Zhao X, Zhang X, Bulović V, Bawendi M, Nelson KA (2017). Terahertz-driven luminescence and colossal stark effect in CdSe-CdS colloidal quantum dots. Nano Lett..

[41] Lu Y, Zhang Q, Wu Q, Chen Z, Liu X, Xu J (2021). Giant enhancement of THz-frequency optical nonlinearity by phonon polariton in ionic crystals. Nat. Commun..

[42] Kozina M, Fechner M, Marsik P, van Driel T, Glownia JM, Bernhard C, Radovic M, Zhu D, Bonetti S, Staub U, Hoffmann MC (2019). Terahertz-driven phonon upconversion in SrTiO_3_. Nat. Phys..

[43] von Hoegen A, Mankowsky R, Fechner M, Först M, Cavalleri A (2018). Probing the interatomic potential of solids with strong-field nonlinear phononics. Nature.

[44] Wu L, Salehi M, Koirala N, Moon J, Oh S, Armitage NP (2016). Quantized Faraday and Kerr rotation and axion electrodynamics of a 3D topological insulator. Science.

[45] Afanasiev D, Hortensius JR, Ivanov BA, Sasani A, Bousquet E, Blanter YM, Mikhaylovskiy RV, Kimel AV, Caviglia AD (2021). Ultrafast control of magnetic interactions via light-driven phonons. Nat. Mater..

[46] Matsuda T, Kanda N, Higo T, Armitage NP, Nakatsuji S, Matsunaga R (2020). Room-temperature terahertz anomalous Hall effect in Weyl antiferromagnet Mn_3_Sn thin films. Nat. Commun..

[47] Vaidya P, Morley SA, van Tol J, Liu Y, Cheng R, Brataas A, Lederman D, Del Barco E (2020). Subterahertz spin pumping from an insulating antiferromagnet. Science.

[48] Siegrist F, Gessner JA, Ossiander M, Denker C, Chang YP, Schröder MC, Guggenmos A, Cui Y, Walowski J, Martens U, Dewhurst JK, Kleineberg U, Münzenberg M, Sharma S, Schultze M (2019). Light-wave dynamic control of magnetism. Nature.

[49] Kampfrath T, Tanaka K, Nelson KA (2013). Resonant and nonresonant control over matter and light by intense terahertz transients. Nat. Photonics.

[50] Ma J, Shrestha R, Adelberg J, Yeh CY, Hossain Z, Knightly E, Jornet JM, Mittleman DM (2018). Security and eavesdropping in terahertz wireless links. Nature.

[51] Sengupta K, Nagatsuma T, Mittleman DM (2018). Terahertz integrated electronic and hybrid electronic–photonic systems. Nat. Electron..

[52] Ummethala S, Harter T, Koehnle K, Li Z, Muehlbrandt S, Kutuvantavida Y, Kemal J, Marin-Palomo P, Schaefer J, Tessmann A, Garlapati SK, Bacher A, Hahn L, Walther M, Zwick T, Randel S, Freude W, Koos C (2019). THz-to-optical conversion in wireless communications using an ultra-broadband plasmonic modulator. Nat. Photonics.

[53] Yang Y, Yamagami Y, Yu X, Pitchappa P, Webber J, Zhang B, Fujita M, Nagatsuma T, Singh R (2020). Terahertz topological photonics for on-chip communication. Nat. Photonics.

[54] Liebermeister L, Nellen S, Kohlhaas RB, Lauck S, Deumer M, Breuer S, Schell M, Globisch B (2021). Optoelectronic frequency-modulated continuous-wave terahertz spectroscopy with 4 THz bandwidth. Nat. Commun..

[55] Khalatpour A, Paulsen AK, Deimert C, Wasilewski ZR, Hu Q (2021). High-power portable terahertz laser systems. Nat. Photonics.

[56] Chen, X., Wang, H., Wang, C., Ouyang, C., Wei, G., Nie, T., Zhao, W., Miao, J., Li, Y., Wang, L., Wu, X.: Efficient generation and arbitrary manipulation of chiral terahertz waves emitted from Bi_2_Te_3_-Fe heterostructures. Adv. Photonics Res. **2**(4), 2000099–2000109 (2021)

[57] Strecker KE, Partridge GB, Truscott AG, Hulet RG (2002). Formation and propagation of matter-wave soliton trains. Nature.

[58] Kimel AV, Kirilyuk A, Usachev PA, Pisarev RV, Balbashov AM, Rasing T (2005). Ultrafast non-thermal control of magnetization by instantaneous photomagnetic pulses. Nature.

[59] Taguchi K, Tatara G (2011). Theory of inverse Faraday effect in a disordered metal in the terahertz regime. Phys. Rev. B Condens. Matter Mater. Phys..

[60] Masson JB, Gallot G (2006). Terahertz achromatic quarter-wave plate. Opt. Lett..

[61] Jia M, Wang Z, Li H, Wang X, Luo W, Sun S, Zhang Y, He Q, Zhou L (2019). Efficient manipulations of circularly polarized terahertz waves with transmissive metasurfaces. Light Sci. Appl..

[62] Chen K, Feng Y, Monticone F, Zhao J, Zhu B, Jiang T, Zhang L, Kim Y, Ding X, Zhang S, Alù A, Qiu CW (2017). A reconfigurable active Huygens’ metalens. Adv. Mater..

[63] Knyazev BA, Choporova YY, Mitkov MS, Pavelyev VS, Volodkin BO (2015). Generation of terahertz surface plasmon polaritons using nondiffractive bessel beams with orbital angular momentum. Phys. Rev. Lett..

[64] Chang CC, Zhao Z, Li D, Taylor AJ, Fan S, Chen HT (2019). Broadband linear-to-circular polarization conversion enabled by birefringent off-resonance reflective metasurfaces. Phys. Rev. Lett..

[65] Xie Z, He J, Wang X, Feng S, Zhang Y (2015). Generation of terahertz vector beams with a concentric ring metal grating and photo-generated carriers. Opt. Lett..

[66] Zhao H, Chen X, Ouyang C, Wang H, Kong D, Yang P, Zhang B, Wang C, Wei G, Nie T, Zhao W, Miao J, Li Y, Wang L, Wu X (2020). Generation and manipulation of chiral terahertz waves in the three-dimensional topological insulator Bi_2_Te_3_. Adv. Photonics.

[67] Sarukura N, Ohtake H, Izumida S, Liu Z (1998). High average-power THz radiation from femtosecond laser-irradiated InAs in a magnetic field and its elliptical polarization characteristics. J. Appl. Phys..

[68] Lu X, Zhang XC (2012). Generation of elliptically polarized terahertz waves from laser-induced plasma with double helix electrodes. Phys. Rev. Lett..

[69] Zhang Z, Chen Y, Cui S, He F, Chen M, Zhang Z, Yu J, Chen L, Sheng Z, Zhang J (2018). Manipulation of polarizations for broadband terahertz waves emitted from laser plasma filaments. Nat. Photonics.

[70] Wang WM, Gibbon P, Sheng ZM, Li YT (2015). Tunable circularly polarized terahertz radiation from magnetized gas plasma. Phys. Rev. Lett..

[71] You YS, Oh TI, Kim KY (2013). Mechanism of elliptically polarized terahertz generation in two-color laser filamentation. Opt. Lett..

[72] Sato M, Higuchi T, Kanda N, Konishi K, Yoshioka K, Suzuki T, Misawa K, Kuwata-Gonokami M (2013). Terahertz polarization pulse shaping with arbitrary field control. Nat. Photonics.

[73] Kanda N, Higuchi T, Shimizu H, Konishi K, Yoshioka K, Kuwata-Gonokami M (2011). The vectorial control of magnetization by light. Nat. Commun..

[74] McDonnell C, Deng J, Sideris S, Ellenbogen T, Li G (2021). Functional THz emitters based on Pancharatnam-Berry phase nonlinear metasurfaces. Nat. Commun..

[75] Seifert TS, Jaiswal S, Barker J, Weber ST, Razdolski I, Cramer J, Gueckstock O, Maehrlein SF, Nadvornik L, Watanabe S, Ciccarelli C, Melnikov A, Jakob G, Münzenberg M, Goennenwein STB, Woltersdorf G, Rethfeld B, Brouwer PW, Wolf M, Kläui M, Kampfrath T (2018). Femtosecond formation dynamics of the spin Seebeck effect revealed by terahertz spectroscopy. Nat. Commun..

[76] Zhou C, Liu YP, Wang Z, Ma SJ, Jia MW, Wu RQ, Zhou L, Zhang W, Liu MK, Wu YZ, Qi J (2018). Broadband terahertz generation via the interface inverse Rashba-Edelstein effect. Phys. Rev. Lett..

[77] Jungfleisch MB, Zhang Q, Zhang W, Pearson JE, Schaller RD, Wen H, Hoffmann A (2018). Control of terahertz emission by ultrafast spin-charge current conversion at Rashba interfaces. Phys. Rev. Lett..

[78] Cheng L, Wang X, Yang W, Chai J, Yang M, Chen M, Wu Y, Chen X, Chi D, Goh KEJ, Zhu JX, Sun H, Wang S, Song JCW, Battiato M, Yang H, Chia EEM (2019). Far out-of-equilibrium spin populations trigger giant spin injection into atomically thin MoS_2_. Nat. Phys..

[79] Zhou X, Song B, Chen X, You Y, Ruan S, Bai H, Zhang W, Ma G, Yao J, Pan F, Jin Z, Song C (2019). Orientation-dependent THz emission in non-collinear antiferromagnetic Mn_3_Sn and Mn_3_Sn-based heterostructures. Appl. Phys. Lett..

[80] Gao Y, Kaushik S, Philip EJ, Li Z, Qin Y, Liu YP, Zhang WL, Su YL, Chen X, Weng H, Kharzeev DE, Liu MK, Qi J (2020). Chiral terahertz wave emission from the Weyl semimetal TaAs. Nat. Commun..

[81] Kong D, Wu X, Wang B, Nie T, Xiao M, Pandey C, Gao Y, Wen L, Zhao W, Ruan C, Miao J, Li Y, Wang L (2019). Broadband spintronic terahertz emitter with magnetic-field manipulated polarizations. Adv. Opt. Mater..

[82] Chen X, Wu X, Shan S, Guo F, Kong D, Wang C, Nie T, Pandey C, Wen L, Zhao W, Ruan C, Miao J, Li Y, Wang L (2019). Generation and manipulation of chiral broadband terahertz waves from cascade spintronic terahertz emitters. Appl. Phys. Lett..

[83] Fang Z, Wang H, Wu X, Shan S, Wang C, Zhao H, Xia C, Nie T, Miao J, Zhang C, Zhao W, Wang L (2019). Nonlinear terahertz emission in the three-dimensional topological insulator Bi_2_Te_3_ by terahertz emission spectroscopy. Appl. Phys. Lett..

[84] Wang B, Shan S, Wu X, Wang C, Pandey C, Nie T, Zhao W, Li Y, Miao J, Wang L (2019). Picosecond nonlinear spintronic dynamics investigated by terahertz emission spectroscopy. Appl. Phys. Lett..

[85] Guo F, Pandey C, Wang C, Nie T, Wen L, Zhao W, Miao J, Wang L, Wu X (2020). Generation of highly efficient terahertz radiation in ferromagnetic heterostructures and its application in spintronic terahertz emission microscopy (STEM). OSA Continuum.

[86] Beaurepaire E, Turner GM, Harrel SM, Beard MC, Bigot JY, Schmuttenmaer CA (2004). Coherent terahertz emission from ferromagnetic films excited by femtosecond laser pulses. Appl. Phys. Lett..

[87] Battiato M, Carva K, Oppeneer PM (2010). Superdiffusive spin transport as a mechanism of ultrafast demagnetization. Phys. Rev. Lett..

[88] Kampfrath T, Battiato M, Maldonado P, Eilers G, Nötzold J, Mährlein S, Zbarsky V, Freimuth F, Mokrousov Y, Blügel S, Wolf M, Radu I, Oppeneer PM, Münzenberg M (2013). Terahertz spin current pulses controlled by magnetic heterostructures. Nat. Nanotechnol..

[89] Seifert T, Jaiswal S, Martens U, Hannegan J, Braun L, Maldonado P, Freimuth F, Kronenberg A, Henrizi J, Radu I, Beaurepaire E, Mokrousov Y, Oppeneer PM, Jourdan M, Jakob G, Turchinovich D, Hayden LM, Wolf M, Münzenberg M, Kläui M, Kampfrath T (2016). Efficient metallic spintronic emitters of ultrabroadband terahertz radiation. Nat. Photonics.

[90] Huisman TJ, Mikhaylovskiy RV, Costa JD, Freimuth F, Paz E, Ventura J, Freitas PP, Blügel S, Mokrousov Y, Rasing T, Kimel AV (2016). Femtosecond control of electric currents in metallic ferromagnetic heterostructures. Nat. Nanotechnol..

[91] Walowski J, Münzenberg M (2016). Perspective: ultrafast magnetism and THz spintronics. J. Appl. Phys..

[92] Wu Y, Elyasi M, Qiu X, Chen M, Liu Y, Ke L, Yang H (2017). High-performance THz emitters based on ferromagnetic/nonmagnetic heterostructures. Adv. Mater..

[93] Seifert T, Jaiswal S, Sajadi M, Jakob G, Winnerl S, Wolf M, Kläui M, Kampfrath T (2017). Ultrabroadband single-cycle terahertz pulses with peak fields of 300 kV⋅cm^−1^ from a metallic spintronic emitter. Appl. Phys. Lett..

[94] Zhang S, Jin Z, Liu X, Zhao W, Lin X, Jing C, Ma G (2017). Photoinduced terahertz radiation and negative conductivity dynamics in Heusler alloy Co_2_MnSn film. Opt. Lett..

[95] Wang X, Cheng L, Zhu D, Wu Y, Chen M, Wang Y, Zhao D, Boothroyd CB, Lam YM, Zhu JX, Battiato M, Song JCW, Yang H, Chia EEM (2018). Ultrafast spin-to-charge conversion at the surface of topological insulator thin films. Adv. Mater..

[96] Feng Z, Yu R, Zhou Y, Lu H, Tan W, Deng H, Liu Q, Zhai Z, Zhu L, Cai J, Miao B, Ding H (2018). Highly efficient spintronic terahertz emitter enabled by metal-dielectric photonic crystal. Adv. Opt. Mater..

[97] Liu C, Wang S, Zhang S, Cai Q, Wang P, Tian C, Zhou L, Wu Y, Tao Z (2021). Active spintronic-metasurface terahertz emitters with tunable chirality. Adv. Photonics.

[98] Niwa H, Yoshikawa N, Kawaguchi M, Hayashi M, Shimano R (2021). Switchable generation of azimuthally- and radially-polarized terahertz beams from a spintronic terahertz emitter. Opt. Express.

[99] Hibberd MT, Lake DS, Johansson NAB, Thomson T, Jamison SP, Graham DM (2019). Magnetic-field tailoring of the terahertz polarization emitted from a spintronic source. Appl. Phys. Lett..

[100] Yang D, Liang J, Zhou C, Sun L, Zheng R, Luo S, Wu Y, Qi J (2016). Powerful and tunable THz emitters based on the Fe/Pt magnetic heterostructure. Adv. Opt. Mater..

[101] Zhang L, Huang Y, Zhu L, Yao Z, Zhao Q, Du W, He Y, Xu X (2019). Polarized THz emission from in-plane dipoles in monolayer tungsten disulfide by linear and circular optical rectification. Adv. Opt. Mater..

